# White-Collar Workers in the Post-Pandemic Era: A Review of Risk and Protective Factors for Mental Well-Being

**DOI:** 10.3390/bs15101313

**Published:** 2025-09-25

**Authors:** Junyi Meng, Lidia Suárez, Chad C. E. Yip, Nigel V. Marsh

**Affiliations:** 1 School of Social and Health Sciences, James Cook University, Singapore 387380, Singapore; junyi.meng@my.jcu.edu.au (J.M.); lidia.suarez@jcu.edu.au (L.S.); chadcheweun.yip@jcu.edu.au (C.C.E.Y.); 2 Tropical Futures Institute, James Cook University, Singapore 387380, Singapore; 3 Margaret Roderick Centre for Mental Health Research, James Cook University, Singapore 387380, Singapore

**Keywords:** workplace stress, employee well-being, turnover intention, white-collar worker, COVID-19, Job Demands-Resources (JD-R) model, occupational health psychology, narrative literature review

## Abstract

This narrative literature review aims to explore the risk and protective factors influencing the mental well-being of white-collar workers in the post-pandemic era. It investigates how factors vary across different phases, including pre-pandemic traditional work models, work-from-home or hybrid models during the pandemic, and the recovery phase of returning to the office in the post-pandemic era. This review highlights the diverse nature of related factors, examining constructs including stress, depression, burnout, thriving, work engagement, workaholism, motivation, workplace civility, and resilience. The Job Demands-Resources model, a recognized theoretical tool for analyzing and understanding the interactions between psychological constructs and their effects on employee well-being and turnover intention, is proposed as a useful framework to consider the relationships between the factors. By synthesizing existing research findings, this review contributes to our understanding of the complex interplay between work-related factors and employee well-being in the evolving landscape of the post-pandemic world. Understanding these dynamics is crucial for developing effective strategies to support white-collar workers’ mental well-being and productivity in the post-pandemic era.

## 1. Introduction

This review covers peer-reviewed studies published in English between 1991 and 2025 that examined the mental well-being of white-collar workers under different work arrangements before, during, and after the COVID-19 pandemic. This review employs a narrative literature review method to increase self-knowledge in this field, provide a comprehensive overview of the dynamic factors influencing workplace mental health, and identify key gaps in research on the mental health of white-collar workers in the post-pandemic era. Unlike systematic reviews or meta-analyses, which focus on quantitative summaries of narrowly defined evidence, this study employs a narrative approach to explore the multifaceted dimensions of workplace mental health and identify key areas requiring further research, thereby providing a foundation for subsequent empirical studies.

The term “white-collar” first appeared in the Logansport Daily Reporter in Indiana in 1910, which referred to non-manual work or workers, such as office work with a clerical, managerial, or administrative nature (Oxford University Press, n.d.). Since the early 20th century, white-collar workers have developed as the current workplace’s mainstay. Many white-collar positions have been created and are common in today’s society concerning government, business, academics, law, healthcare, finance, engineering, and information technology. In the modern work life of white-collar workers, they are suffering from permanent connectivity, instantaneous communication, heavy workloads, multitasking, interruptions by immediate information, expectations of accessibility, and constant changes and updates in hardware and software causing long term connectivity, which are contributing to their stress and extending their work time (Ayyagari et al., 2011). This concept has become a foundational classification in social science research, widely employed to analyze occupational structures and working conditions in industrialized societies.

The impact of COVID-19 on the workplace in recent years has led to new aspects of workplace stressors for white-collar workers. For example, the work-from-home model, flexible work model, and hybrid work model depend on the application of technology tools, which increases technostress among white-collar workers (Harkiolakis & Komodromos, 2023; Jamal et al., 2024; Lathabhavan & Griffiths, 2024; Pflügner et al., 2021). Also, the instability of the work environment is a significant factor in aggravating feelings of work stress. According to available research, it has been noted that some people feel stressed when they work from home (Al Riyami et al., 2024; Newman et al., 2022), while some feel stressed when they return to their regular workplace order (Fan & Moen, 2023; Pandita et al., 2024). Furthermore, the crisis in the economy caused by COVID-19 is widespread and persistent, which means that the economic downturn in the post-epidemic era can be a stressor for white-collar workers (Massar et al., 2023; Wallberg et al., 2023). Thus, in the weakened workplace caused by COVID-19, there appears to be an increase in workaholics as employees tend to have more work to do, and longer working hours are expected of them (Venkatesh et al., 2021).

Employee well-being and turnover intention are crucial outcomes for assessing the mental health of white-collar workers. Firstly, well-being represents a dynamic state enabling individuals to develop their potential, work effectively and creatively, build positive and strong relationships, and contribute to society (Venkatesh et al., 2021). Employees are likely to achieve greater well-being if they are not compelled to overwork and compromise their health due to job demands set by their supervisors, managers, and organizations (Harkiolakis & Komodromos, 2023). In addition, life satisfaction, stress, psychological strain, and loneliness are recognized as various mental well-being measures (Levacher et al., 2023). Findings from well-being research indicate that employee well-being is negatively influenced by crisis, personality, work motivation, work environment, occupation, place of residence, and organizational actions (Levacher et al., 2023; Newman et al., 2022). Optimal employee well-being requires a flexible workplace, harmonious social relations, and a state of happiness (Barath & Schmidt, 2022; Henke et al., 2022; Newman et al., 2022; Qamar et al., 2023). Secondly, turnover intention refers to an employee’s desire to leave their current job, which may reflect their level of job satisfaction (Radu et al., 2023). Compared to the traditional work model, the flexible work model has been found to reduce employee turnover levels (Çemberci et al., 2022; Harkiolakis & Komodromos, 2023; L. Y. Wang & Xie, 2023). Moreover, Jamal et al. (2024) indicated that employees’ turnover intentions can be forestalled and reduced by decreasing job demands, providing support, and developing personal resources. Additionally, working in a positive and collaborative environment can reduce employee turnover intentions (Harkiolakis & Komodromos, 2023). However, higher turnover intentions were closely related to burnout caused by job demands (Jamal et al., 2024), and achieving high productivity at work may come at the cost of increased turnover intentions (Venkatesh et al., 2021).

In predicting the factors influencing employee well-being and turnover intention, the Job Demands-Resources (JD-R) model plays an essential role, as it is a leading occupational stress model for examining how job and personal characteristics affect worker outcomes (Schaufeli & Taris, 2014). Also, based on the different outcomes produced by related factors, those that lead to adverse effects can be classified as risk factors. In contrast, those that result in positive effects can be defined as protective factors.

This literature review will first introduce the Job Demands-Resources (JD-R) model as the theoretical framework. Building upon this foundation, it will then explore findings from studies examining common stress issues white-collar workers encounter across different work patterns. Finally, it reviews the constructs identified to explore factors influencing white-collar workers’ mental well-being and turnover intention in the post-pandemic era.

## 2. Job Demands-Resources Model

The JD-R model was first introduced at the beginning of this century (Demerouti et al., 2001). It is an integrated conceptual framework for assessing workplace environments (Schaufeli, 2017). Initially, the dimensions of the JD-R model only encompassed job demands and job resources (Demerouti et al., 2001). Demerouti et al. (2001) defined job demands as “aspects of the job that require sustained physical or mental effort and are therefore associated with certain physiological and psychological costs” and defined job resources as “aspects of the job that may do any of the following: (a) be functional in achieving work goals; (b) reduce job demands and the associated physiological and psychological costs; (c) stimulate personal growth and development.” According to the JD-R model theory created by Demerouti et al. (2001), job demands include physical workload, time pressure, recipient contact, physical environment, and shift work. Job resources include feedback, rewards, job control, participation, job security, and supervisor support.

Over time, the JD-R model has evolved to include individual dimensions such as personal demands and personal resources (Bakker & Demerouti, 2017; Marathe et al., 2019). Bakker and Demerouti (2017) expanded the original, simple JD-R model by incorporating the individual’s role in modifying the effects of job demands and resources on energy and motivation, in the form of job crafting, personal resources, and self-undermining. Marathe et al. (2019) extended the JD-R model by developing a conceptual model of various psychological work states centered on job thrust and personal thrust. This aimed to offer a nuanced understanding of work states and propose a comprehensive nomological network to explain job-related demands and resources, personal demands and resources, and various work behaviors and attitudes (Marathe et al., 2019).

**Figure 1:**
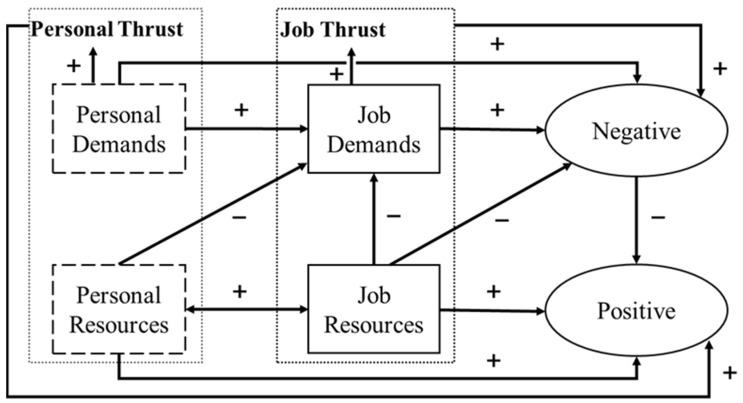
Job Demands-Resources Model.

Figure 1 illustrates the updated JD-R Model, including the latest developments in JD-R theory, and shows the interactions between job demands, job resources, personal demands, and personal resources.

The JD-R model can help analyze and understand the relationships between different psychological constructs regarding job demands, job resources, personal demands, and personal resources. As seen in Figure 1, job demands can lead to negative outcomes, while job resources can lead to positive outcomes. Additionally, job resources can reduce job demands and negative outcomes. On the other hand, personal demands can increase both job demands and negative outcomes. Regarding personal resources, both personal and job resources mutually promote each other, enhance positive outcomes, and reduce job demands.

## 3. Changes in the Workplace and Their Impact on White-Collar Workers

Using the outbreak of the COVID-19 pandemic as a key time point, this review aims to clarify how factors related to white-collar workplaces have changed and what effects they have had before and after the pandemic, presented through a historical narrative.

### 3.1. Method

This narrative literature review aims to explore the risk and protective factors influencing mental well-being among white-collar workers. The methodology for this review followed the SANRA (a scale for the quality assessment of narrative review articles) guidelines (Baethge et al., 2019), with its six criteria addressed as follows:

(1) Importance—This review is of great significance to all stakeholders concerned with workplace stress. It may inform relevant interventions, which in turn may help improve workers’ well-being and reduce turnover.
(2) Aims of the review—The primary objective is to synthesize existing evidence on risk factors and protective factors, identifying temporal changes in white-collar workplaces in the context of COVID-19.
(3) Literature search—The Web of Science database was used as the main data source.
(4) Referencing and presentation: This paper refers and presents relevant literature by categorizing it into three groups: pre-COVID-19 pandemic (Table 1), during-COVID-19 pandemic (Table 2), and post-COVID-19 pandemic (Table 3).
(5) Evidence—Research design and purpose are included.
(6) Relevant endpoint data—Key findings are included.

This search was conducted using the Web of Science database. For the pre-pandemic period, the search terms included “Work stress OR workaholism” + “White-collar worker” + “Burnout OR Thriving” and were limited to articles published before the outbreak of the COVID-19 pandemic (prior to 2020). This search resulted in the inclusion of 17 relevant articles as references. For research published after the outbreak of COVID-19, the search focused on the keywords “Work stress OR workaholism in a post-pandemic world” + “Burnout OR Thriving” + “Factor”, with articles restricted to those published within the last 5 years. Ultimately, 30 papers were included in the review for this period. Overall, 47 related papers were reviewed in this section. We found that including “white-collar worker” as a term in our search of the literature published after the outbreak of COVID-19 (2020+) substantially influenced the scope of the results from the second search. Perhaps due to the narrow timeframe, only a few relevant papers were found. Therefore, to ensure a comprehensive search, we excluded the term and instead manually reviewed the papers found for those that used white-collar workers as participants. To further ensure the comprehensiveness of the search, we also identified additional seminal publications through backward citation tracing.

This review employed a three-stage analytical approach to synthesize the literature. First, we applied the Job Demands-Resources model as our theoretical framework to analyze evolving workplace demands and resources. Second, we compared findings through cross-study synthesis tables to identify consistent patterns across temporal phases (pre-pandemic, pandemic, post-pandemic) and work arrangements (office, remote, hybrid). Finally, we identified research gaps through critical reflection on study limitations and theoretical inconsistencies. This combined deductive-inductive approach enabled a comprehensive interpretation of white-collar workers’ mental well-being throughout workplace transformations.

### 3.2. Findings from Pre-COVID-19 Studies

This section will highlight the factors contributing to lower levels of mental well-being among white-collar workers in the pre-COVID-19 period.

Evidence from previous studies shows that an uncivil work environment can lead to a wide range of negative consequences. As reported in a Turkish survey, workplace bullying can increase stress levels among employees, and it found that 55% of respondents admitted to having experienced various forms of bullying, and 47% reported witnessing such behaviour (Bilgel et al., 2006). Research on Canadian white-collar workers revealed that various professional roles could significantly influence their well-being (Bhalla et al., 1991). Specifically, clerical workers reported a heightened sense of insufficiency, which refers to inadequacy or not meeting job expectations (Bhalla et al., 1991). Middle-level officers, such as supervisors and team leads, experienced elevated workplace conflicts, including disagreements with colleagues and challenges in managing subordinates (Bhalla et al., 1991). These officers also reported notably lower job satisfaction and organizational loyalty (Bhalla et al., 1991). In contrast, managers exhibited a heightened sense of responsibility towards others, indicating their increased concern for the welfare and performance of their team members (Bhalla et al., 1991). In addition, the impact of work time on the mental health of white-collar workers is notable, as demonstrated by the findings of a two-year follow-up study conducted among Japanese white-collar workers (Hino et al., 2019). This study highlighted that reducing overtime work hours could prevent mental health deterioration (Hino et al., 2019). According to Bourbonnais et al. (1996), there exists a clear correlation between work-related stress and psychological distress among white-collar professionals.

Apart from the outcomes caused by the white-collar workplace environment, such as workplace incivility, job dissatisfaction, organizational conflict, a heightened sense of insufficiency, elevated levels of conflict, and extensive work hours, other significant factors can also influence the mental health of white-collar professionals. Specifically, work stress can also lead to various health problems for individuals. Tsai (2012) conducted a health status study of white-collar migrant workers, and found that their hyperlipidemia and self-reported neck pain were related to significantly lower physical health and functioning. Additionally, gastric ulcers, cardiovascular disease, and poor sleep were correlated with decreased mental health and psychological well-being. Tsai (2012) discovered that perceived work-related stress and depression indirectly affected the health-related quality of life among these workers.

Moreover, some studies have shown a positive relationship between job stress and the use of psychotropic drugs among white-collar workers. For example, Moisan et al. (1999) indicated that drug use increased as job strain increased, and social support had no impact on drug use (Laflamme et al., 1998). Another study also highlighted that the stressful work environment of white-collar workers led to an increased utilization of prescribed psychotropic drugs (Morissette & Dedobbeleer, 1997). In a study examining the factors associated with depression among white-collar workers, Nakamura et al. (2011) discovered that depression in this group was closely linked to several key factors. These included personal traits such as perfectionism and high self-expectations, low self-esteem, and external psychological pressures such as demanding workloads, lack of job security, and workplace conflicts (Nakamura et al., 2011). This study emphasized the complex interplay between individual characteristics and environmental stressors in contributing to depression among white-collar workers (Nakamura et al., 2011).

Other research has indicated that gender could independently predict certain variables related to white-collar workers’ health conditions and job satisfaction. For example, Renault Moraes et al. (1993) found that women reported higher levels of job stress than men. Lindfors et al. (2006) indicated that men were more likely to experience job dissatisfaction due to the lack of career advancement opportunities. Lundberg et al. (1994) also found that women had higher rates of work–life conflict, impacting their overall well-being. However, educational level could not independently predict stress-related outcomes (Renault Moraes et al., 1993), while job type could predict job satisfaction (Renault Moraes et al., 1993). For example, Renault Moraes et al. (1993) found that managerial positions were associated with the highest levels of job satisfaction compared to clerical or administrative roles.

Table 1 summarizes the papers reviewed for the pre-COVID-19 period (before 2020). These focused on the traditional work-from-office model.

**Table 1:** Summary of Studies on the Pre-COVID-19 Work Model.

Author	Design	Purpose	Key Findings
Bhalla et al. (1991)	Cross-sectional study	To examine various stressors at work and outside work.	Among work stressors, conflict, ambiguity, and insufficiency were most closely tied to vocational outcomes.
Bilgel et al. (2006)	Cross-sectional study	To determine the prevalence of reported workplace bullying among a group of white-collar workers, to assess its impact on health, and to evaluate the effectiveness of workplace support for bullied employees.	White-collar workers who experienced workplace bullying reported higher levels of anxiety, depression, and overall lower mental well-being.
Bourbonnais et al. (1996)	Cross-sectional study	To assess if workers under high job strain, defined as high psychological demands and low decision latitude, experience more psychological distress than those not under high job strain.	Workers facing high job strain, characterized by high demands and low control, suffer more psychological distress than those with lower job strain.
Hagihara et al. (1998)	Cross-sectional study	To evaluate the relative importance of work and non-work factors in deciding the subject’s job satisfaction level.	The results suggest improving job satisfaction is more effectively achieved through company policies, such as clear communication, professional development, fair compensation, and a supportive work environment, rather than individual efforts.
Hagihara et al. (2000)	Cross-sectional study	To assess after-work drinking interactions with coworkers and work stressor variables among white-collar workers.	The findings suggest that after-work drinking with coworkers alleviated job dissatisfaction, but only for those with lower levels of work stress.
Hansen et al. (2010)	Longitudinal study	To investigate the link between physical activity and perceived job demand, job control, stress, energy, and cortisol levels (morning and evening) in saliva among white-collar workers.	Physically active employees perceive less stress and more energy. Vigorous physical activity influenced the relationship between stress, energy, and salivary cortisol.
Hino et al. (2019)	Longitudinal study	To examine the impact of changes in overtime hours on depressive symptoms among white-collar workers.	Reducing overtime hours may protect mental health.
Kageyama et al. (2001)	Cross-sectional study	To identify the factors influencing sleep debt on weekdays among Japanese white-collar workers.	Sleep debt on weekdays in Japanese white-collar workers was positively associated with age, overtime, and self-rated workload.
Lindfors et al. (2006)	Cross-sectional study	To study the relationship between total workload (paid and unpaid) and psychological well-being and symptoms in full-time employed women and men.	Among full-time white-collar workers, gender differences were significant. Increased unpaid work hours decreased self-acceptance and environmental mastery in women, while paid work increased personal growth but decreased life goals. Men reported more personal growth in paid work.
Lundberg et al. (1994)	Cross-sectional study	To assess various aspects of paid and unpaid forms of productive activity among white-collar workers.	Women report more stress and work conflict than men, especially those aged 35–39 with children. Men enjoy more workplace autonomy. Higher management experiences less conflict and more control.
Moisan et al. (1999)	Cross-sectional study	To assess the link between high psychological demand, low decision latitude at work, and the use of psychotropic drugs among white-collar workers.	Job strain may determine psychotropic drug use among white-collar workers, but workplace social support does not seem to affect this link.
Renault Moraes et al. (1993)	Cross-sectional study	To study occupational stress in a medium-sized government organization.	Younger employees and women experienced the highest levels of occupational stress.
Morissette and Dedobbeleer (1997)	Cross-sectional study	To hypothesize the contribution of specific professional factors to women’s long-term use of prescribed psychotropic drugs.	Results suggest that a high-stress work environment leads to increased prescribed drug consumption.
Nakamura et al. (2011)	Cross-sectional study	To clarify the factors that lead to depression.	There are close relationships between depression in white-collar workers and their characteristics, feelings of self-esteem, and psychological stress experienced outside rather than inside the workplace.
Sandmark and Renstig (2010)	Qualitative study	To understand impaired work ability, leading ultimately to long-term sick leave.	Work and private life factors were crucial in the informants’ deteriorating health and long-term sick leave. Job mismatches, company profitability issues, and poor leadership led to stress-related symptoms and decreased work capacity.
Tokuyama et al. (2003)	Longitudinal study	To identify risk factors for depression among workers.	Major depression in white-collar workers is associated with job stress, insufficient social support, extended work hours, and poor work–life balance.
Tsai (2012)	Cross-sectional study	To explore health-related quality of life and work-related stress and their risk factors.	Perceived work-related stress and depression indirectly influence health-related quality of life by mediating the link between job demands and health outcomes.

During the pre-COVID-19 period, research was based on the traditional office work model, identifying some common issues. Factors such as work overload, high job demands, lack of physical activity, and unfair compensation contributed to chronic work stress among white-collar workers, revealing an increased risk of cardiovascular disease, a compromised immune system, heightened anxiety, depression, and burnout. Additionally, changes in coping mechanisms and behaviours, such as increased alcohol consumption and decreased physical activity, were also observed. Furthermore, job strain and conflicts led to higher turnover intention (Bhalla et al., 1991; Bourbonnais et al., 1996). As the post-pandemic work environment and order for white-collar workers are returning to the traditional office setting, understanding the characteristics and workplace factors of the traditional office model is valuable. This understanding is crucial to examine why and how these factors have changed post-COVID-19 compared to pre-COVID-19, and therefore, to develop strategies that support well-being in the evolving work landscape.

### 3.3. Findings from Post-COVID-19 Studies

During the initial outbreak of COVID-19, the traditional office work pattern of white-collar workers shifted to the remote work model due to lockdown policies and health concerns. This shift sparked a newfound interest among scholars in studying workplace stress among white-collar workers in this context. Compared to the conventional work model, remote work, swiftly popularized after the outbreak of COVID-19, relied heavily on advanced technology for implementation. Lathabhavan and Griffiths (2024) reported positive associations between the simplicity of technology utilization, managerial encouragement, and peer assistance with employees’ self-efficacy and a negative correlation between self-efficacy and technological stress. Significant positive correlations were also observed between self-efficacy, training transfer, work engagement, and job satisfaction (Lathabhavan & Griffiths, 2024).

Another study pointed out that various factors influence the work behaviour of white-collar workers, including age, which can affect productivity and adaptability; attitude, which impacts motivation and commitment; income, which influences job satisfaction and performance; perceived behavioral control, which affects the ability to handle job demands; work–life balance, which influences stress levels and job satisfaction; and the level of interpersonal contact, which impacts teamwork and communication (Han et al., 2024). In low-risk scenarios, such as the pre-COVID-19 work environment, preferences for traditional office work or telecommuting were consistent with these factors. However, in high-risk scenarios, such as during the COVID-19 pandemic, preferences shifted due to health concerns and safety measures (Han et al., 2024). Adapting to telecommuting during COVID-19 was smoother for those already inclined toward it but more challenging for others (Han et al., 2024). Moreover, adopting hybrid and remote work presented opportunities and challenges (Chafi et al., 2022). On the positive side, it offered autonomy, flexibility, enhanced individual performance, and improved work–life balance (Chafi et al., 2022). However, it also brought challenges, such as feelings of isolation and a decline in camaraderie among colleagues (Chafi et al., 2022). The impact of flexible work arrangements during the COVID-19 pandemic was also two-fold. From the perspective of job demand, flexible work arrangements increased white-collar workers’ role ambiguity, which in turn reduced their innovative performance; from the standpoint of job resources, flexible work arrangements increased white-collar workers’ psychological empowerment, which in turn enhanced their innovative performance (L. Y. Wang & Xie, 2023). From the perspective of individual differences, those with high role breadth self-efficacy could enhance psychological empowerment and reduce role ambiguity under a variable work system, thereby promoting their innovative performance (L. Y. Wang & Xie, 2023).

A study targeting the Indian white-collar population working from home found that longer remote work, increased professional experience, and a support function role were associated with a higher probability of accomplishing the same or more significant workload (Kumar et al., 2023). Conversely, being female or married decreased this probability, while being employed in manufacturing or services showed no significant impact. Regarding psychological factors, a stronger desire for autonomy reduced the likelihood of maintaining or increasing the workload at home (Kumar et al., 2023). For personal and professional resources, engaging in job crafting to enhance structural job resources and receiving supervisor support were associated with an increased likelihood of accomplishing the same or greater workload at home than in the office (Kumar et al., 2023).

Nevertheless, the lockdown action did not directly lead to burnout outcome. Although there was a short-term decrease in life satisfaction among white-collar workers following the lockdown’s implementation, their stress and psychological strain were alleviated after the second week of restrictions (Levacher et al., 2023). Personality traits such as introversion, neuroticism, agreeableness, and conscientiousness were strong predictors for burnout dimensions despite the COVID-19 pandemic having little impact on the development of employee burnout syndrome (Rodriguez-Lopez & Rubio-Valdehita, 2021). Moreover, knowledge employees, represented by white-collar workers, could thrive in a remote work environment if managed with empathy, granted ample flexibility and autonomy by organizations, and provided with opportunities for professional development and social interaction during the COVID-19 Lockdown Era (Harkiolakis & Komodromos, 2023).

During remote work among white-collar workers under the background of COVID-19, the factors affecting the work behaviour and mental health of white-collar workers differed somewhat from those before the pandemic. Parental responsibilities and marital status have emerged as significant determinants impacting work engagement among flexible work arrangements (Çemberci et al., 2022). Factors like familial and peer disruptions, individual capacity to handle stress, and limitations in resources could affect work-from-home performance (R. Wang et al., 2022). Behaviour changes in terms of increased sleep duration and decreased physical activities of employees were long-lasting (Massar et al., 2023). Moreover, concerning the mental well-being of white-collar workers, findings indicated that factors such as demand, management, relationship, and support were negatively correlated with mental health outcomes (Borrelli et al., 2023). The changes in work circumstances due to the COVID-19 pandemic significantly decreased ratings on situational stability, accompanied by an enhanced positive impact of conscientiousness on productivity (Venkatesh et al., 2021). During the COVID-19 pandemic, the relationship between conscientiousness, stress, and contentment underwent a notable shift. Individuals with higher levels of conscientiousness, typically associated with positive outcomes, reported experiencing increased stress and decreased contentment. This reversal was partly due to the heightened job requirements and extended working hours imposed by the pandemic conditions. As a result, highly conscientious individuals found themselves under greater pressure to meet elevated demands, leading to increased stress levels. Additionally, the prolonged working hours contributed to a decrease in overall contentment, as the balance between work and personal life became more challenging to maintain (Venkatesh et al., 2021).

Table 2 summarizes the papers reviewed for the COVID-19 period, which focused on the work-from-home model.

**Table 2:** Summary of Studies on the During-COVID-19 Work Model.

Author	Design	Purpose	Key Findings
Al Riyami et al. (2024)	Cross-sectional study	To understand how the emotions of employees are affected by work-from-home measures.	The protective effect of mindfulness on reducing workplace ostracism is stronger with moderate to high organizational support but not when support is low.
Barath and Schmidt (2022)	Cross-sectional study	To discuss the changes employers will apply regarding the work environment and office layout.	The findings suggest that an increasingly mobile workforce and expanding new work styles may not necessarily result in an office exodus. Still, they will undoubtedly lead to more flexible and reduced office utilization.
Çemberci et al. (2022)	Cross-sectional study	To examine how marital status, job experience, and having children affect work engagement in white-collar workers with flexible hours.	There is a significant positive relationship between job experience and work engagement. Additionally, job satisfaction, organizational support, and work–life balance significantly influence work engagement.
Chafi et al. (2022)	Qualitative study	To identify needs, challenges, and sustainability potential in remote and hybrid work.	Individuals gain flexibility but struggle with boundaries. Groups benefit from digital tools but face reduced cohesion. Leaders can innovate but must ensure resource equity.
Costin et al. (2023)	Systematic literature review	To analyze evidence on how remote employees without consistent organizational support during COVID-19 faced increased job demands, strain, low satisfaction, and burnout.	Severe psychological symptoms and stress were linked to poor organizational communication and rising workload.
Henke et al. (2022)	Qualitative study	To identify factors influencing success in remote work.	Regular mindfulness practice among employees significantly reduces workplace stress and improves overall job satisfaction and productivity.
Jamal et al. (2024)	Longitudinal study	To explore the adverse employee outcomes of working from home.	Employees experienced increased job demands during COVID-19.
Kohn et al. (2025)	Mixed study	To learn about employees’ digital resilience from enforced remote work transitions.	Digital resilience in employees hinges on organizational support, technical resources, and their ability to utilize communication technologies effectively.
Kumar et al. (2023)	Cross-sectional study	To study factors affecting the amount of work done at home.	Longer work-from-home exposure, greater work experience, and support roles increased the likelihood of maintaining or increasing home work.
Lathabhavan and Griffiths (2024)	Cross-sectional study	To investigate the role of technology, manager support, and peer support on self-efficacy and job outcomes of employees while working from home.	The study demonstrated the importance of social persuasion (including technology) while working from home in enhancing employees’ self-efficacy and job outcomes.
Levacher et al. (2023)	Longitudinal study	To investigate longitudinal changes in mental well-being during the first lockdown of the COVID-19 pandemic.	Personality traits significantly impact mental well-being. Key traits such as extraversion, conscientiousness, and emotional stability were particularly important in determining overall mental health outcomes.
Plester and Lloyd (2023)	Qualitative study	To explore how workplace fun and psychological safety impact a positive work environment.	Hybrid work can lead to greater interpersonal ambiguity due to the lack of embodied cues in virtual interactions. This absence of non-verbal communication, such as body language and facial expressions, can result in misunderstandings and reduced team cohesion.
Radu et al. (2023)	Cross-sectional study	To understand remote work’s impact on employee perception, psychological safety, and job performance is crucial for organizations.	When psychological safety is high, the positive relationship between employee sentiment around remote work and work performance is stronger.
Rodriguez-Lopez and Rubio-Valdehita (2021)	Cross-sectional study	To assess how sociodemographic variables, pandemic concern, and personality predict burnout.	Personality significantly affects workers’ pandemic-related job concerns and burnout development.
Uzkurt et al. (2025)	Cross-sectional study	To explore the effect of government support on employees’ job performance and motivation perceptions.	Government support positively impacts employees’ motivation and job performance.
Venkatesh et al. (2021)	Quasi-experimental study	To test the moderating effect of situational strength.	COVID-19-induced changes in situational strength may increase burnout, dissatisfaction, and turnover risk among conscientious employees if not managed well.
L. Y. Wang and Xie (2023)	Cross-sectional study	To explore how flexible work arrangements impact employee innovation performance.	Flexible work arrangements increase role ambiguity and reduce innovation but also boost psychological empowerment and enhance it.
Y. Z. Wang and Yu (2023)	Cross-sectional study	To identify spousal support factors affecting female knowledge workers’ work-from-home outcomes and willingness.	The findings could explain how a husband’s support can improve his wife’s well-being when working from home.
R. Wang et al. (2022)	Longitudinal study	To understand the impact of factors on work-from-home behavior.	Remote work significantly enhances job satisfaction and productivity, especially when employees receive adequate organizational support and resources.

In summary, the widespread adoption of telework during COVID-19 has led to new research examining its impact on white-collar professionals. Key findings indicate that telework, increased digital reliance, blurred work–life boundaries, and reduced social interactions significantly affect their mental health, with studies focusing on stress levels, mental well-being, mindfulness practice, mental stability, and work–life balance challenges.

Furthermore, the post-COVID-19 period is based on the return-to-office phase for white-collar workers as a typical example of re-establishing traditional work routines (Gibson et al., 2023; Pandita et al., 2024) and is an essential stage in the recovery of conventional work patterns after the outbreak of COVID-19. By analyzing dimensions of well-being, burnout, job satisfaction, life satisfaction, and work–life conflict, one study reported that returning to a traditional work model could heighten employees’ stress levels (Fan & Moen, 2023). Increased emotional exhaustion, work-family conflicts, and absenteeism have also become prominent factors contributing to the stress of returning to the office (Pandita et al., 2024).

Table 3 summarizes the papers reviewed for the post-COVID-19 period, which focused on the return-to-office work model.

**Table 3:** Summary of Studies on the Post-COVID-19 Model.

Author	Design	Purpose	Key Findings
Fan and Moen (2023)	Longitudinal study	To comprehend the structured dynamics of subjective well-being linked with changing workplaces.	Cluster analysis revealed four distinct patterns of well-being based on burnout, work–life conflict, and satisfaction with job and life.
Gibson et al. (2023)	Qualitative study	To investigate the effects of returning to office work on employee productivity, mental health, and overall job satisfaction following the COVID-19 pandemic.	Returning to office work improved employee productivity but had mixed effects on mental health and job satisfaction, highlighting the need for flexible work options.
Han et al. (2024)	Quantitative study	To determine appropriate candidates for telework and to specify which types of workers are best suited for various levels of telework, establish scientifically sound and reasonable hybrid work ratios and procedures, and assess their suitability.	Regular remote work increases employee autonomy and job satisfaction but can also lead to feelings of isolation and decreased team cohesion.
Harkiolakis and Komodromos (2023)	Integrative literature review	To explore the impact of hybrid work models on employee engagement and organizational performance, focusing on identifying best practices for implementation.	Knowledge workers thrive in hybrid environments when given empathy, autonomy, flexibility, social interaction opportunities, and professional advancement by organizations.
Krajcik et al. (2023)	Cross-sectional study	To ascertain the preferences of employees from culturally diverse backgrounds regarding their work location and schedule following the conclusion of the pandemic.	The suggested hybrid work model appears to be the most fitting solution according to employees’ preferences.
Liang et al. (2023)	Cross-sectional study	To examine the link between employees’ expected company-allowed telecommuting levels and their preferred frequency post-pandemic.	A discrepancy was found between the telecommuting frequency companies are expected to allow and the frequency preferred by employees.
Massar et al. (2023)	Longitudinal study	To study the impact of working from home on sleep and activity patterns during the later stages of the COVID-19 pandemic transition to normalcy.	Sleep and physical activity changes during the pandemic persisted into later stages, suggesting long-term effects. Efforts are needed to maximize benefits and reduce drawbacks.
Pandita et al. (2024)	Qualitative study	To investigate the aspects of return-to-office stress and how organizations can assist employees in managing it.	Hybrid work models significantly enhance work–life balance and job satisfaction but require robust digital infrastructure and clear communication strategies for optimal effectiveness.
Qamar et al. (2023)	Mixed study	To examine how high-performance work systems enhance happiness through the sequential pathways of career aspiration and thriving at work.	The study findings reveal a positive relationship between high-performance work systems and career aspiration.
Borrelli et al. (2023)	Cross-sectional study	To study the outcomes of anxiety and depression traits in workers under stress-related work.	High demands negatively impacted mental health, while strong management, support, and positive relationships were associated with improved mental health outcomes.
Wallberg et al. (2023)	Qualitative study	To examine the long-term effects of remote work on employee collaboration, innovation, and organizational culture in various industries.	Pandemic-induced changes in working conditions caused difficulties for young employees and managers when flexibility was insufficient.

As shown in Table 3, the workplace environment has changed significantly after the outbreak of COVID-19. Despite attempts to restore the original practices, potential risks persist. Fundamental changes include an increased emphasis on remote and hybrid work models, which have improved employee autonomy and job satisfaction but also led to feelings of isolation and decreased team cohesion. Studies highlight the mixed effects of returning to office work environments on mental health and job satisfaction, underscoring the need for flexible work options. Additionally, there is a greater focus on balancing productivity with well-being, addressing work–life conflict, and providing reliable technology and clear communication strategies. Understanding these shifts is important for developing strategies to support employee well-being and reduce their turnover intention in the evolving work landscape.

In short, the focus of occupational health psychology research has varied over different periods of the pandemic. Since the outbreak of COVID-19, new work models such as work-from-home, telecommuting, and hybrid arrangements have become prevalent in research. Consequently, workplace civility and employee resilience in crises have also gained prominence. Work stress, depression, work engagement, and burnout continue to be classic constructs explored across the various phases of the COVID-19 pandemic. Recent research directions include exploring the challenges and advantages of telework settings, examining coping strategies in virtual work environments, evaluating the role of digital connectivity in exacerbating or mitigating workplace stress, and assessing the long-term effects of these shifts on individual health and productivity. Additionally, there is growing interest in how organizations can promote employees’ physical and mental health in telework settings through flexible scheduling, virtual mental health resources, and a healthy work–life balance.

## 4. Occupational Health Psychology in the Post-Pandemic World

An understanding of white-collar worker well-being in the post-pandemic world requires the integration of constructs that have come to prominence since the outbreak of COVID-19 with relevant pre-COVID-19 common constructs related to white-collar workplace stress, resignation propensity, and overwork. These factors include stress, depression, burnout, thriving, work engagement, workaholism, motivation, workplace civility, and resilience (Table 4). Consideration of all these factors may help fill the research gap in understanding the mental well-being of white-collar workers in the evolving post-pandemic landscape.

### 4.1. Stress

Work-related stress (Borrelli et al., 2023; Harkiolakis & Komodromos, 2023; Willeke et al., 2021) is also known as job strain (Bourbonnais et al., 1996; Laflamme et al., 1998), or work distress (Borrelli et al., 2023; Çemberci et al., 2022) in the existing literature. Although these terms are synonymous and describe stress experienced by white-collar workers in the workplace, there are subtle differences when they are compared. Specifically, work-related stress tends to have a broader scope than the other two terms, emphasizing overall stress experienced within the workplace. According to Bourbonnais et al. (1996), combining high psychological demand and low decision latitude could increase employee stress and negative health outcomes. Moreover, work distress focuses more on negative emotions resulting from stressors (Borrelli et al., 2023).

In general, the causes of work-related stress usually include work overload (Costin et al., 2023; Hino et al., 2019), role ambiguity (L. Y. Wang & Xie, 2023), job insecurity (Costin et al., 2023), lack of support (Al Riyami et al., 2024; Costin et al., 2023), interpersonal relationships (Bilgel et al., 2006), gender (Morissette & Dedobbeleer, 1997; Y. Z. Wang & Yu, 2023), personal characteristics (Rodriguez-Lopez & Rubio-Valdehita, 2021), and the work environment (Chafi et al., 2022).

### 4.2. Depression

The feeling of depression among white-collar workers is a possible negative consequence of job stress (Willeke et al., 2021). Multiple factors contribute to the development of depression in this group (Tokuyama et al., 2003). Conditions such as workplace ostracism (Al Riyami et al., 2024), work–life imbalance (Massar et al., 2023), low levels of psychological security (Radu et al., 2023), personality type, low self-esteem, and psychological stress experienced outside the workplace (Nakamura et al., 2011) can all lead to depressive symptoms. Moreover, studies have also indicated a correlation between employees’ depression and the geographic culture in which they live. Willeke et al. (2021) found that societal attitudes toward mental health, social support, and regional economic conditions affect the level of depression among employees. In brief, depression represents a severe adverse outcome caused by stress, posing significant risks to the physical and mental well-being of employees (Tokuyama et al., 2003) and, in extreme cases, may lead to suicide (Kato, 2014).

### 4.3. Burnout

Burnout results from stressors and is characterized by a chronic state of physical and emotional exhaustion, mental distancing, and decreased personal efficacy (Harkiolakis & Komodromos, 2023; Pflügner et al., 2021). Burnout in the workplace consists of negative feelings toward one’s occupation and a sense of personal accomplishment deficiency (Harkiolakis & Komodromos, 2023). A previous study found that employed white-collar workers experienced higher burnout levels than self-employed individuals due to rigid work structures, less autonomy, and higher job demands and pressures (Willeke et al., 2021).

Factors associated with job burnout include techno-stressors (Krajcik et al., 2023; Pflügner et al., 2021), excessive working hours (Venkatesh et al., 2021), high level of employee responsibility (Venkatesh et al., 2021), personality type, insomnia, anxiety, depression, uncertainty, negative future expectations (Rodriguez-Lopez & Rubio-Valdehita, 2021), increased workload (Costin et al., 2023), less social connection (Costin et al., 2023), negative emotional behavior (Costin et al., 2023), and gender role (Fan & Moen, 2023).

Furthermore, some studies have identified solutions that reduce the sense of burnout at work. For example, one study reported that psychological empowerment could serve as a protective factor against burnout (L. Y. Wang & Xie, 2023). In response to techno-stressors, providing technology and technical support can help reduce some aspects of burnout (Krajcik et al., 2023). Similarly, burnout-related feelings can be relieved by turning off cameras and microphones (Newman et al., 2022).

Job burnout can adversely affect the physical and mental health as well as the job performance of white-collar workers. In terms of personal health, it can lead to sleep disturbance, state anxiety, acute stress, and derealization symptoms (Costin et al., 2023). In terms of job performance, it can lead to workplace disturbances, low productivity (Costin et al., 2023), and turnover intention (Venkatesh et al., 2021).

### 4.4. Thriving

Work stress does not always lead to negative outcomes but can also enable employees to thrive (Venkatesh et al., 2021). Work-related thriving refers to a state of flourishing and fulfillment in the workplace. It is characterized by a collective experience of learning and vitality and can enhance employees’ psychological well-being (Qamar et al., 2023). A previous study found that career aspirations were positively associated with thriving at work, and thriving at work was positively associated with happiness in the workplace (Qamar et al., 2023).

Thriving at work is driven by factors such as professional achievement, career growth, and progress toward career goals (Qamar et al., 2023). In this context, employees consistently devote effort to their work and exhibit high energy for learning and handling tasks (Qamar et al., 2023). Studies indicate that thriving at work can positively impact employees’ subjective health, job satisfaction, and work commitment (Kleine et al., 2019) while reducing well-being concerns (Qamar et al., 2023). Overall, thriving at work plays a mediating role, significantly mediating the relationships between employee well-being and workplace safety (Okros & Virga, 2023).

### 4.5. Work Engagement

Work engagement is a positive, fulfilling, work-related state of mind characterized by high vigor, dedication, and absorption (Lathabhavan & Griffiths, 2024). It represents a positive, emotional-motivational state where individuals exhibit high energy, commitment, and focused attention toward their work activities (Çemberci et al., 2022). However, excessive work engagement is viewed as workaholism, which is considered a negative phenomenon. Also, work engagement is regarded as a relevant measure of job outcomes (Lathabhavan & Griffiths, 2024), reflecting employees’ enthusiastic involvement and commitment to their work tasks.

Work engagement can be influenced by various factors, including workplace ostracism (Al Riyami et al., 2024), the sense of belonging and being valued (Al Riyami et al., 2024), social support (Kumar et al., 2023), marital and parental status (Çemberci et al., 2022), as well as performance feedback, personal skills, autonomy, learning opportunities (Çemberci et al., 2022), and the feelings of job stress and burnout (Costin et al., 2023). Additionally, research suggests that work attitudes, such as work engagement, may have been more challenging during the COVID-19 pandemic because of increased stress, disrupted work schedules, isolation, and remote work models (Newman et al., 2022).

### 4.6. Workaholism

Workaholism is defined initially as the behavior of a person whose desire for work is so excessive that it noticeably disrupts or interferes with the individual’s health and well-being, interpersonal relationships, and social functioning (McMillan et al., 2001). Those who exhibit this phenomenon are called “workaholics” (Clark et al., 2016). The development of workaholism is associated with achievement-oriented personality traits exemplified by perfectionism and Type A personality but it is generally independent of other personality and demographic variables (Clark et al., 2016). Currently, most scholars view workaholism as an addiction to work, characterized by an inner obsessive–compulsive disorder toward work (Clark et al., 2016; McMillan et al., 2001).

Though workaholics devote much time and energy to work, they are not productive workers due to the higher health and well-being risks (Clark et al., 2016). Specifically, workaholism is not only associated with negative organizational and individual variables but also linked to negative outcomes such as work stress, burnout, low job satisfaction, poor well-being, and work–life conflict (Clark et al., 2016).

### 4.7. Motivation

Motivation refers to the mental processes that provide purpose and direction for one’s behavior, an internal drive to satisfy a need, and the willingness to achieve a goal (Uzkurt et al., 2025). Motivation is a prerequisite for encouraging employees to accomplish their work tasks effectively (Uzkurt et al., 2025). The prevailing view in the literature suggests that the primary motivators include feelings of self-fulfillment, achievement, meaningfulness, and opportunities for advancement (Uzkurt et al., 2025). Additionally, a previous meta-analysis identified expectancy, goal-setting, and self-efficacy as crucial dimensions to measure motivation (Judge & Ilies, 2002).

Psychological needs such as autonomy, competence, and relatedness can influence individuals’ motivations and lead to various outcomes (Kumar et al., 2023). Furthermore, research indicates that factors such as perceived organizational support (Al Riyami et al., 2024), work flexibility (Massar et al., 2023), personal conscientiousness (Venkatesh et al., 2021), and economic assistance (Uzkurt et al., 2025) have a positive impact on work motivation. Conversely, factors such as personal frustration (Kumar et al., 2023), role ambiguity (L. Y. Wang & Xie, 2023), anxiety emotions (Newman et al., 2022), and perceptions of crisis strength (Newman et al., 2022) negatively influence work motivation.

As far as work motivation itself is concerned, it can help boost employee productivity (Kumar et al., 2023) and improve job performance, though at the cost of higher stress and lower satisfaction (Venkatesh et al., 2021).

### 4.8. Workplace Civility

“Workplace civility” is a collective concept referring to a civilized work environment characterized by respect, consideration, and courtesy (Di Fabio & Gori, 2016). Although the term workplace civility is not exclusively used in much of the published research, descriptions of beneficial organizational behaviors adequately capture the above characteristics.

Workplace civility is also highly valued in the return-to-office model. Gibson et al. (2023) suggested that assessment of workplace civility or culture should consider factors such as the form of address between colleagues, response time and speed of work messages, kindness, empathy, quality of work delivered, and general social manners. Workplace civility is closely related to the physical and mental health of employees. For example, employees may feel pressured to meet unrealistic job demands if an organization’s culture emphasizes overtime and workload excessively (Pandita et al., 2024). On the contrary, a culture of good workplace civility can lead to many positive outcomes. For example, it may increase work satisfaction, enhance engagement, and reduce stress and burnout (Di Fabio & Gori, 2016).

### 4.9. Resilience

Resilience is the capacity to bounce back from setbacks in the face of multiple adversities, and it has significant implications for managing crisis scenarios (Newman et al., 2022). Scholars define resilience as a positive adjustment that focuses on recovery from threatening circumstances and inspires individuals to grow, adapt to changes, and persist through challenges (Jamal et al., 2024; Kohn et al., 2025). Resilience is closely related to robustness, reliability, sustainability, coping, healing, recovery, and psychological toughness (Kohn et al., 2025). Furthermore, it encompasses four dimensions: technical resilience, organizational resilience, social resilience, and economic resilience (Kohn et al., 2025). Technological resilience refers to the robustness of systems; organizational resilience refers to the adaptability of firms; social resilience refers to the coping capacity of communities and individuals; and economic resilience refers to the economy’s durability (Kohn et al., 2025). Organizational resilience helps companies support employees, while social resilience helps individuals manage stress and realize work–life balance (Kohn et al., 2025).

Overall, resilience is considered a positive psychological resource (Radu et al., 2023), and it has been found to moderate the relationship between turnover intention and burnout (Jamal et al., 2024). Specifically, resilient employees exhibit a lower intention to leave, even though they feel burned out (Jamal et al., 2024).

Table 5 presents a comparative analysis of key psychological constructs distinguishing pre- and post-COVID-19 work environments.

Conceptualizing and comparing existing research findings on these factors may provide a theoretical basis for further refining their roles in determining mental well-being among white-collar workers in the post-COVID-19 pandemic era. Combining the abovementioned factors related to the mental well-being of white-collar workers, the following hypotheses can be derived from the JD-R model: In the JD-R model, stress due to job demands is associated with negative outcomes, including burnout, depression, and increased turnover intention. Personal demands, such as workaholism, can also lead to these effects. Conversely, job resources like workplace civility help lessen job demands’ impact, reduce stress and burnout, and improve overall well-being. Job resources and personal resources are closely linked to thriving at work and can foster positive outcomes like motivation, resilience, and work engagement.

## 5. Conclusions

By adopting a narrative approach, this literature review identified the risk and protective factors associated with mental well-being in white-collar workers. Findings from the existing research showed that these related factors are diverse and vary, particularly in light of individual personality differences. Additionally, shifts in work models resulting from the COVID-19 crisis exerted an impact on the relevant variables of stress, depression, burnout, thriving, work engagement, workaholism, motivation, workplace civility, and resilience. The impact of these psychological constructs and their interrelationships can best be understood within the framework of the Job Demands-Resources Model. Work patterns shifted from traditional office models to work-from-home or hybrid models and now are transitioning back to the return-to-office model. These shifts in work models have influenced both the workplace environment and individual employees. Consequently, the findings presented here provide a basis for delving into the post-pandemic factors within white-collar worker settings and hold significant innovative value, as they can help identify crucial strategies for enhancing mental well-being and reducing turnover intention in an ever-changing work environment.

## Figures and Tables

**Table 4:** Definitions of Key Psychological Constructs.

Stress	Psychological responses to workplace demands beyond coping capacity
Depression	Negative effects of work stress, persistent moodiness
Burnout	Chronic syndrome from prolonged stress: exhaustion + distancing + reduced efficacy
Thriving	Maintaining a positive combination of learning and energy despite the pressures of the situation
Work Engagement	Positive work state with vigor, dedication, and absorption
Workaholism	Compulsive work addiction
Motivation	Internal drive directing work behavior
Workplace Civility	A work environment characterized by respect and courtesy
Resilience	Ability to adapt to and recover from adversity

**Table 5:** Constructs Before and After COVID-19.

Constructs	Shifts From Pre- to Post-COVID	JD-R Model Alignment
Stress	Physical → Digital stressors	New job demands (technology); Reduced personal resources (boundary control)
Depression	Traditional → Instable mode	Personal demands (adaptation); Resource deficiency (social support)
Burnout	Contextual shift from office → virtual exhaustion	Chronic job demands; Fewer recovery resources
Thriving	Formal → informal development pathways	New job resources (flexibility); Enhanced personal resources (autonomy)
Work Engagement	Fixed → flexible workplace/time	Resource transformation (digital tools enable new engagement modes)
Workaholism	Observable → invisible overwork	Job demands (accessibility); Personal demands (self-regulation) collision
Motivation	Organizational → personal value	Personal resources (autonomy) gain prominence
Workplace Civility	Embodied → mediated interactions	Job resources now include digital social capital
Resilience	Static trait → dynamic skill set	Personal resources expansion (adaptability); New job resources (flexibility)

## Data Availability

No new data were created or analyzed in this study. Data sharing is not applicable to this article.
